# Improved Predictive Tools for Structural Properties of Metal–Organic Frameworks

**DOI:** 10.3390/molecules25071552

**Published:** 2020-03-28

**Authors:** Indrani Choudhuri, Donald G. Truhlar

**Affiliations:** Department of Chemistry, Chemical Theory Center, and Minnesota Supercomputing Institute, University of Minnesota, Minneapolis, MN 55455-0431, USA; indranimoni@gmail.com

**Keywords:** density functional theory, metal-organic frameworks, structural properties, lattice constant, pore sizes, bond lengths, bond angles

## Abstract

The accurate determination of structural parameters is necessary to understand the electronic and magnetic properties of metal–organic frameworks (MOFs) and is a first step toward accurate calculations of electronic structure and function for separations and catalysis. Theoretical structural determination of metal-organic frameworks is particularly challenging because they involve ionic, covalent, and noncovalent interactions, which must be treated in a balanced fashion. Here, we apply a diverse group of local exchange-correlation functionals (PBE, PBEsol, PBE-D2, PBE-D3, vdW-DF2, SOGGA, MN15-L, revM06-L, SCAN, and revTPSS) to a broad test set of MOFs to seek the most accurate functionals to study various structural aspects of porous solids, in particular to study lattice constants, unit cell volume, two types of pore size characteristics, bond lengths, bond angles, and torsional angles). The recently developed meta functionals revM06-L and SCAN, without adding any molecular mechanics terms, are able to predict more accurate structures than previously recommended functionals, both those without molecular mechanics terms (PBE, PBEsol, vdW-DF2, and revTPSS) and those with them (PBE-D2 and PBE-D3). To provide a broader test, these two functionals are also tested for lattice constants and band gaps of unary, binary, and ternary semiconductors.

## 1. Introduction

Metal–organic frameworks (MOFs) are microporous and mesoporous solids in which inorganometallic nodes (which may be, for example, metal cations or metal oxide clusters) are connected with organic linkers [1,2,3]. MOFs have applications and potential applications for gas separation and storage [4,5,6], energy storage [7,8], photocatalysis [9,10,11], and other catalytic [12,13,14] processes. Theory and computation are useful tools to predict the accurate structures of MOFs for the precise understanding of their functional behavior.

Kohn-Sham density functional theory (KS-DFT) is the most popular way to calculate structural parameters of solids [15,16,17,18,19]. The accuracy of a KS-DFT calculation of a system depends on the choice of exchange-correlation (XC) functional. Our study builds on previous work by Sholl and co-workers [20], who studied a set of 12 MOFs containing various metal atoms. Of six functionals tested, they found that the PBE-D2, PBE-D3, and vdW-DF2 functionals are most promising for the accurate determination of the structural parameters of the MOFs (lattice parameters, unit cell volume, bonded parameters, and pore descriptors). Because improved functionals have become available since the work of Sholl et al., the present work considers the same set of 12 MOFs (listed in Table 1) studied by Sholl et al. [20] and two other MOFs (UiO-66 and MOF-5 or IRMOF-1, which were added to increase the diversity of the test set) with ten functionals—the three that they found to perform best and seven others. We study several structural parameters (lattice constants, pore size, bond length, bond angle, torsional angle, and unit cell volume), and we consider four kinds of functionals:

- GA functionals: gradient approximations in which the energy density depends on the local spin-specific electron densities and their gradients. In particular, we consider PBE [21,22], PBEsol [23], and SOGGA [24] in this group.
- Meta functionals in which the energy density also depends on the spin-specific local kinetic energy densities. In particular, we consider MN15-L [25], revM06-L [26], SCAN [27], and revTPSS [28] in this group.
- GA+MM functionals in which a GA is augmented with molecular mechanics terms. In particular, we consider PBE-D2 and PBE-D3 in this group [29,30].
- A functional with nonlocal correlation, vdW-DF2 [31], which has local exchange and nonlocal correlation.

Because nonlocal Hartree-Fock exchange is very expensive in plane wave codes, most calculations on solids are restricted to using only local exchange (sometimes called semilocal exchange) in which the exchange energy density at a point is approximated in terms of functions evaluated at that point, and both the work of Sholl and coworkers and the present work consider only functionals with local exchange. All functionals considered here except vdW-DF also have local correlation, but an efficient algorithm [32] is available for vdW-DF so the cost is not impractically higher than that of the local functionals. In particular, we found that the relative costs for the calculations reported here with the different kinds of functionals are approximately: GA, 1; GA+MM, 1.2, meta, 1.5–2, and vdW-DF2, 3.5). 

Table 1 includes the reference code from the Cambridge Structural Database (CSD) and CoRE MOF database [47] of the crystal structure associated with each MOF in the test set. There are nine different metal centers among the twelve MOFs; this includes two lanthanides (Dy and Sm). We considered two different oxidation states of Cu (Cu^+1^ and Cu^+2^) and Fe (Fe^+2^ and Fe^+3^) (Table 1), and both magnetic and non-magnetic MOFs are considered in the study. 

We also note the work of Tran et al. [48,49], who studied lattice constants, cohesive energies, and bulk moduli of strongly and weakly bound solids and found the best behavior for rev-VDW-DF2 and various meta functionals. The reader is directed to their studies for information complementary to the present study.

## 2. Computational Methods

We performed all the density functional theory calculations with the latest version (5.4.4) of the Vienna Ab initio Simulation Package (VASP) [50,51] with periodic boundary conditions. The core-valence electron interactions were described by using the projector augmented wave (PAW) method [52]. The kinetic energy cutoff was 520 eV for all calculations (the same as used by Sholl and coworkers [20]). All the lattice constants, cell shapes, and atomic coordinates were fully relaxed during the optimizations. The C_6_ and R_0_ parameters for PBE-D2 method for elements H–Xe are the original values [29] where available, but for Dy and Sm we used C_6_ = 33.98 Jnm^6^/mol and R_0_ = 2.226 Å, as used by Sholl and co-workers [20]. We set a convergence criterion of 10^−5^ eV for electronic energy minimization and a convergence criterion of 0.0002 eV/Å on the force on each atom. Monkhorst–Pack *k*-point grids [53] were used as specified in Table S1 (tables with a prefix S are in the Supplementary Materials).

The Cu_3_H_4_C_10_O_10_ (MURCEH) MOF requires additional considerations. We used a 2 × 1 × 1 supercell because the unit cell contains an odd number of metal centers (3 Cu centers); the supercell is used to make an even number of metal centers (6 Cu centers) in order to study the magnetic ground state. All the structural parameters of MURCEH are calculated based on the 2 × 1 × 1 supercell.

We performed spin-polarized KS-DFT calculations to determine the magnetic ground states. The initial guesses of the number of unpaired electrons on metal centers and the magnetic ground states are listed in Table S1. The magnetic properties of the MOFs are determined based on the number of unpaired electrons in the metal center of the ground state and are listed in Table S1. We used the PBEsol and PBE-D3 functionals to verify the magnetic ground states, and the magnetic ground states of the MOFs determined in our calculations agree well with the experimental results, as well as with the work of Sholl and co-workers [20].

Using the Zeo++ software [54,55,56], we calculated two kinds of pore sizes, in particular the pore limiting diameter (PLD) and the largest cavity diameter (LCD). The PLD is defined as the diameter of the largest sphere that can move through the MOF. The LCD is defined as the diameter of the largest sphere that can fit in the pore of the MOFs. According to the Zeo++ software website [57], the LCD and PLD are designated as “Di” and “Dif”.

To assess the performance of the functionals on the various properties, we calculated the mean signed error (MSE), mean unsigned error (MUE), and mean unsigned percentage error (MUPE) for each combination of functional and property. The errors of the structural parameters (lattice parameters, lattice angles, bond lengths, bond angles, and torsional angles) are defined as the deviations between experimental results and the results calculated by DFT.

We also studied unary, binary, and ternary semiconductors with two of the functionals (revM06-L and SCAN). For these calculations, we used a kinetic energy cutoff of 520 eV, a 12 × 12 × 12 Monkhorst–Pack *k*-point mesh, and the same convergence criteria and force criteria as for the MOFs.

## 3. Results and Discussion

### 3.1. Lattice Constants and Unit Cell Volume

**Figure 1:**
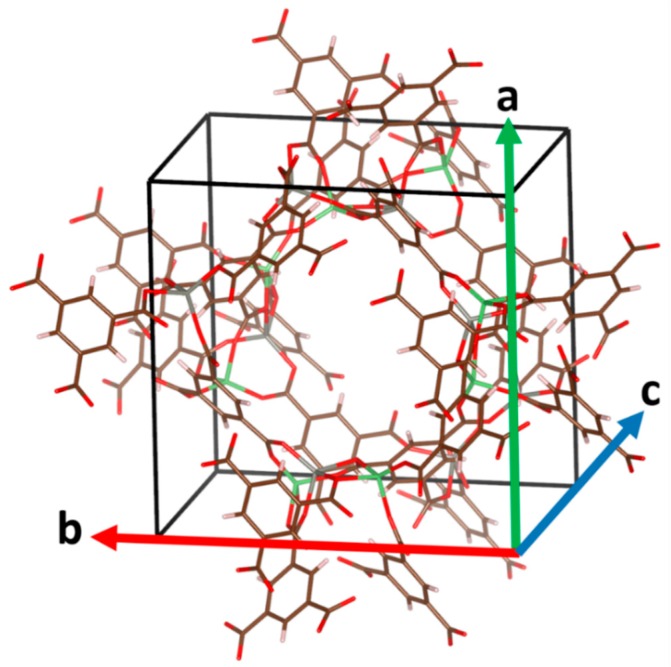
Experimental structure of Li_8_Zn_8_H_24_C_72_O_48_ MOF (reference code: WAJJAU). The unit cell is bounded by black lines. The directions in which the lattice constants *a*, *b*, and *c* are measured are shown as green, red, and blue arrows, respectively.

The first and most important structural parameters of a solid are the lattice constants. The three lattice constants are designated as *a*, *b*, and *c* (see Figure 1).

To ensure that all the crystals are of high quality, the experimental lattice constants of the 14 MOFs are from solvent-free and disorder-free single-crystal X-ray diffraction (XRD) data at room temperature (290–310 K) with an *R*-factor (the measure of the agreement between the experimental X-ray diffraction data and the crystallographic model, also known as residual factor, reliability factor, *R*-value, or *R*_Work_) of less than 10 [58].

**Figure 2:**
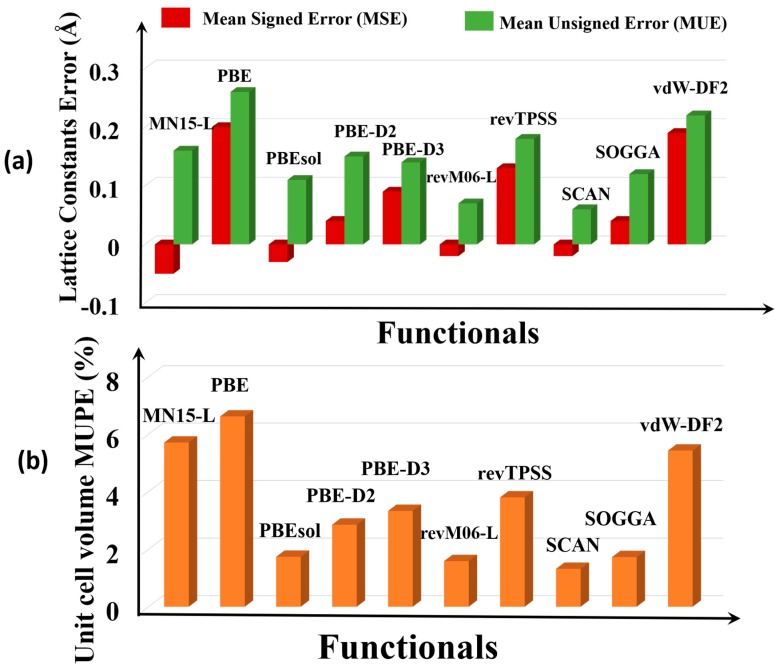
Errors in (**a**) lattice constants and (**b**) unit cell volumes.

Figure 2a shows the error statistics of the various functionals for predicting lattice constants. The MSEs show that on average, PBE, PBE-D3, vdW-DF2, and revTPSS overestimate the lattice constants, and PBEsol, PBE-D2, SOGGA, MN15-L, revM06-L, and SCAN underestimate them. The SCAN (0.06 Å) and revM06–L (0.07 Å) functionals are the most accurate (i.e., have the smallest MUE). The PBE functional has the largest MUE, which is consistent with what is usually found for PBE for other kinds of solids.

Full details given in the Supplementary Materials show that the maximum deviation of the PBE functional is for Ag_4_C_12_Cl_4_O_8_ MOF (RORQOE) (*a* = 0.50 Å, *b* = 0.28 and *c* = 0.98 Å), which also agrees well with the results of Sholl and co-workers [20]. However, SCAN and revM06-L have errors in the lattice constants of this MOF that are 5–24 times smaller. SCAN and revM06-L are very accurate for the lattice constants of Cd_12_H_48_C_72_N_72_O_48_ MOF (GUPCUQ01); the deviation is only 0.01 Å for both functionals (Table S2). We find that SOGGA and PBEsol are almost equally good for predicting the lattice constants of the MOFs, with MUEs of 0.11 Å for PBEsol and 0.12 Å for SOGGA, although PBEsol has an empirical parameter adjusted to lattice constants, and SOGGA does not. The revM06-L and SCAN functionals are even better and have the lowest MUEs of the functionals studied here.

We also studied the changes in lattice angles of the unit cells of the test sets of MOFs. We found that there is no effect of the functionals on the lattice angles for seven of the MOFs, but there are some changes in lattice angles for the rest of the MOFs. Based on those changes we have calculated the MSE, MUE, and MUPE (given in Table S8) statistics, and these values show that SCAN and revM06-L perform best for this property; both have an MUPE of only 0.3%.

Unit cell volume is another measure of structure. Figure 2b shows that SCAN (1.3 %) and revM06-L (MUPE = 1.6%) are the most accurate functionals for unit cell volume, whereas PBEsol (1.7 %) and SOGGA (1.7 %) have slightly larger MUPEs, and PBE (6.6 %), MN15-L (5.7 %), PBE–D3 (3.3 %), and vdw-DF2 (5.4%) greatly overestimate unit cell volume. Full details from which these error statistics were calculated are in Table S3.

### 3.2. Pore Size

The accurate prediction of pore diameters is critical for understanding separations and catalytic selectivity. Here, we have studied two different kinds of pore diameters of the MOFs; the LCD and PLD nomenclature (explained in Section 2) is taken from the work of Haldoupis and coworkers [59].

**Figure 3:**
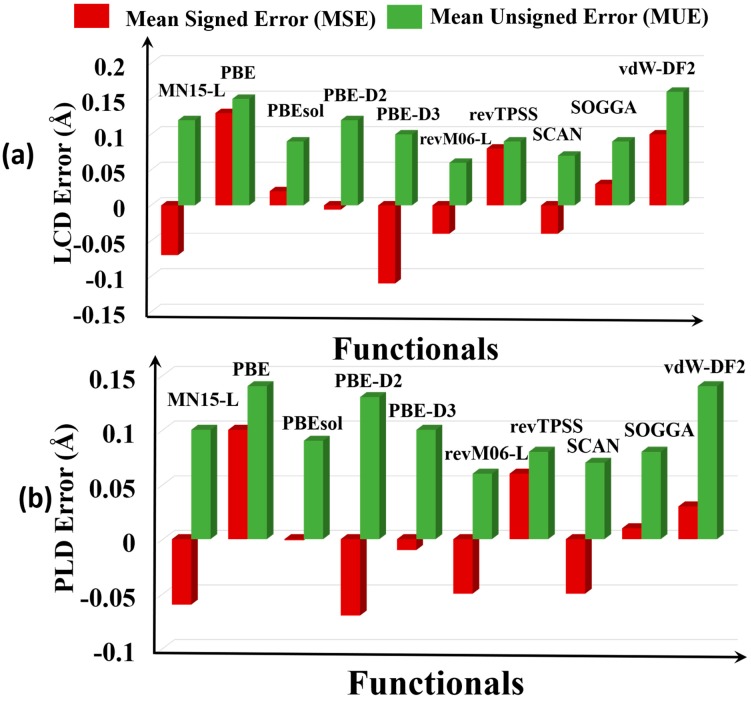
Graphical representation of the errors of pore sizes: (**a**) largest cavity diameters (LCDs) and (**b**) pore limiting diameters (PLDs).

We found that PBE, PBEsol, SOGGA, and revTPSS overestimate both measures of pore size (Figure 3), and PBE-D2, PBE-D3, MN15-L, revM06-L, and SCAN underestimate the LCD and PLD pore sizes of the MOFs. The MUEs show that the most accurate functional for pore sizes is revM06-L, with an MUE of 0.06 Å for PLD and LCD. The SCAN functional is the second best with MUEs of 0.07–0.08 Å. PBE and vdW-DF2 and MN15-L have the highest errors, with MUEs in the range 0.13–0.15 Å, about twice as large as for revM06-L and SCAN.

### 3.3. Bond Lengths

Bond length is the distance between two bonded atoms. We only considered the bond lengths of the inorganic nodes. We considered 60 bond lengths (Table S5) in the 14 MOFs; among the 60 bond lengths, 2 are metal–chlorine bonds (M–Cl), 6 are metal–nitrogen bonds (M–N), and the other 52 bonds are metal–oxygen bonds (M–O). The kinds of bonds included in the tests are Li-O, Fe-O, Cu-O, Zn-O, Zr–O, Ag-O, Cd-O, Sm-O, Dy-O, Co-N, Cd-N, Cu-Cl, and Ag-Cl.

**Figure 4:**
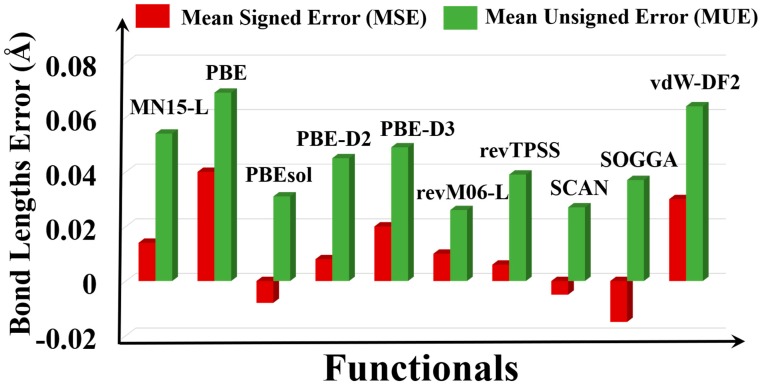
Graphical representation of the errors of bond lengths of the MOFs with respect to different functionals.

The PBE and vdW-DF2 functionals highly overestimate the bond lengths (Figure 4); we found that some of the Ag–O, Cd–O, and Fe–O bond lengths are overestimated by 0.10 Å by PBE (Table S5). The overestimated bond lengths by PBE and vdW-DF2 are consistent with their overestimation of the lattice constants (Table S2). The Cu–N bond in Cu_8_H_8_C_8_N_12_C_l8_ (QEJZUB01) is highly overestimated by 0.23 Å by PBE, as also found by Sholl et al. [20]. The Fe–O bond in Fe_4_H_4_C_4_O_12_ (DEMLIR) also has large errors for some functionals: 0.23 and 0.18 Å by MN15-L and SOGGA respectively, and this large deviation of the Fe–O bond lengths is consistent with the high lattice constant errors for this system (Table S2). Overall, revM06-L has the smallest MUE (0.026 Å) and SCAN has the second smallest error (0.027 Å).

### 3.4. Bond Angles and Torsional Angles

**Figure 5:**
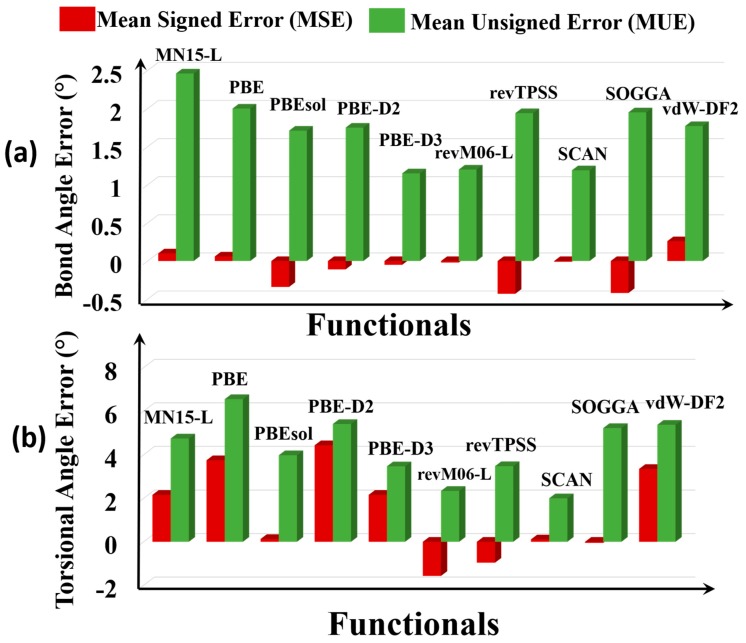
Errors of (**a**) bond angles and (**b**) torsional angles.

A bond angle is the angle between two geminal bonds. We consider 80 bond angles, all in the nodes—in particular 60 O–M–O angles, 8 N–M–N angles, 4 O–M–Cl angles, 4 M–O–M angles, and 2 each of N–M–Cl and O–M–N angles. The kinds of bond angles included in the tests are O-Ag-O, O-Cd-O, O-Cu-O, O-Fe-O, O-Zn-O, O–Zr–O, O-Dy-O, O-Sm-O, O-Li-O, O-Cd-N, N-Co-N, N-Cd-N, O-Ag-Cl, N-Cu-Cl, Li-O-Zn, Zn–O-Zn, and Zr–O-Zr. The error statistics are in Figure 5a.

The Ag-O-Cl bond angle of Ag_4_C_12_Cl_4_O_8_ MOF (RORQOE) and the O-Sm-O bond angle in Sm_2_H_12_C_10_O_14_ MOF (KOMJEC) are subject to particularly large errors. Overall, MN15-L (2.7°) has the largest MUE among all the functionals studied, and SCAN (1.3°) and revM06-L (1.3°) are the most accurate for this property.

A torsional angle is the angle between the ABC plane and the BCD plane in an A–B–C–D bond sequence. We studied 14 torsional angles on the nodes. The torsions included in the tests are Ag-O-Ag-Cl, Cu-O-Cu-O, Li-O-Zn-O, Cu-Cl-Cu-Cl, Fe-O-P-O, O-Sm-O-C, O-Dy-O-C, O-Fe-O-C, O-Zn-O-C, N-Cd-N-C, O-Cd-N-C, N-Co-N-C, Zn-O-Zn-O, and Zr-O-Zr-O. The error statistics are in Table S7.

On average, PBE, PBE-D2, PBE-D3, vdW-DF2, MN15-L, SCAN, PBEsol, and SOGGA overestimate the torsional angles (Figure 5b), and revM06-L and revTPSS underestimate them. PBE has the highest MUE (6.6°) among all the functionals; this large MUE is dominated by large errors for Co_2_C_8_N_12_ (HAWVOQ01), Ag_4_C_12_C_l4_O_8_ (RORQOE), Sm_2_H1_2_C_10_O_14_ (KOMJEC), and Cu_3_H_4_C_10_O_10_ (MURCEH). Among all the functionals, SCAN (MUE = 2.0°) most accurately predicts the torsional angles of the MOFs, and revM06-L is the second best (MUE = 2.3°).

### 3.5. Broader Test on Semiconductors

**Figure 6:**
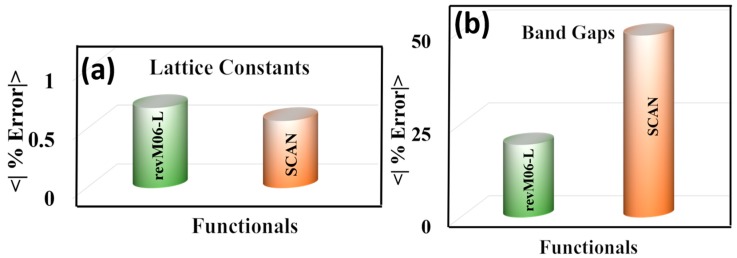
Mean unsigned percentage errors (MUPE) of (**a**) lattice constants and (**b**) band gaps of semiconductors.

The results presented above show that the revM06-L and SCAN functionals give the most accurate results for the structural parameters of MOFs. Since the performance of these two functionals for structural properties of MOFs is similar, we carried out a broader test of these functionals for important crystalline properties, in particular for lattice parameters and band gaps of ten semiconductors, namely Si, Ge, SiC, β-GaN, CdS, CdSe, ZnS, ZnO, TiO_2_–anatase, and TiO_2_–rutile. The results are in Figure 6 and Table S8. This comparison shows that revM06-L and SCAN both are accurate to better than 0.7% for predicting the lattice constants of the semiconductors, but revM06-L is much more accurate for predicting the band gaps of the semiconductors.

Previous work in the literature has shown that the band gaps of semiconductors are most accurately determined by hybrid functionals (e.g., HSE06 [60]) and by high-local-exchange functionals (e.g., HLE17 [61,62]). For large systems like MOFs, HLE17 is as accurate as HSE06 with almost 100 times less computational cost [61,62], and for this reason we focus here on local functionals. The previous study showed that HLE17 is far more accurate for predicting the band gaps of unary and binary semiconductors (Si, Ge, SiC, β-GaN, CdS, CdSe, ZnS, and ZnO) than local functionals like PBE, PBE+U with *U* set to 4.0 eV, PBEsol, B3LYP, TPSS, GAM, and HCTH/407, but the lattice constants are not accurate [61]. A similar trend was found in our recent paper where HLE17 gave more accurate band gaps for TiO_2_ (both anatase and rutile) than other local functionals like PBE, PBEsol, and PBE+U (U = 4.0 eV) [62], but HLE17 did not produce good structural parameters. In contrast, the present study shows that the revM06-L and SCAN local functionals are both able to produce good structural parameters. There is, however, still a trade-off in that neither of these functionals produces band gaps as accurate as those calculated by HLE17. The key finding of the present section, though, is that the trade-off is less severe with revM06-L than with SCAN, because revM06-L yields more accurate band gaps than SCAN.

## 4. Conclusions for MOFs

We studied the structural parameters–lattice parameters, unit cell volume, bond lengths, bond angles, torsional angles, and pore sizes (largest cavity diameters and pore limiting diameters) of a diverse set of 14 MOFs with nine local functionals and one nonlocal functional. The mean unsigned percentage errors (MUPEs) for the various properties are summarized in Table 2. The last column of the table averages these MUPEs to get an average MUPE (AMUPE) to provide a more global summary of the present findings. The three functionals previously [20] found to be most accurate have AMUPEs of 3.0–3.5%. Five of the functionals introduced in the present study have smaller AMUPEs than that, namely SCAN (1.5%), revM06-L (1.7%), PBEsol (2.4%), SOGGA (2.5%), and revTPSS (2.5%). Thus, these functionals, especially SCAN and revM06-L, are highly recommended for future work on the computational design of MOFs.

## Figures and Tables

**Table 1:** List of metal–organic frameworks (MOFs) studied here along with their reference codes, metal centers, oxidation states, space groups, and experimental references.

MOF ^a^	Reference Code ^b^	Metal	Oxidation State of Metal	Space Group	Reference ^c^
Ag_4_C_12_Cl_4_O_8_	RORQOE	Ag	I	P 21/c	[33]
Cd_12_H_48_C_72_N_72_O_48_	GUPCUQ01	Cd	II	P 1	[34]
Cd_2_H_10_C_16_N_4_O_10_	PIJGEV	Cd	II	P 1	[35]
Zn_1_H_4_C_4_O_4_	OFUWIV01	Zn	II	C2	[36]
Li_8_Zn_8_H_24_C_72_O_48_	WAJJAU	Li, Zn	Li (I), Zn (II)	P4(1)2(1)2	[37]
Co_2_C_8_N_12_	HAWVOQ01	Co	II	P 1	[38]
Cu_3_H_4_C_10_O_10_	MURCEH	Cu	II	P 1	[39]
Cu_8_H_8_C_8_N_12_Cl_8_	QEJZUB01	Cu	I and II	P 1	[40]
Dy_2_H_12_C_12_N_2_O_16_	YORSII	Dy	III	P 1	[41]
Fe_4_H_4_C_4_O_12_	HOGWAB	Fe	II	P 1	[42]
Fe_4_P_4_H_16_C_8_O_24_	DEMLIR	Fe	III	P 1	[43]
Sm_2_H_12_C_10_O_14_	KOMJEC	Sm	III	P 1	[44]
Zr_24_O_128_C_192_ (UiO-66)	RUBTAK	Zr	IV	P 1	[45]
Zn_32_O_104_C_192_H_96_ (MOF-5)	SAHYIK	Zn	II	P 1	[46]

*^a^* Chemical formula of the unit cell of the MOF; *^b^* The reference code associated with each structure in the Cambridge Structural Database (CSD) and CoRE MOF database [47]; *^c^* Experimental reference.

**Table 2:** Mean unsigned percentage errors (MUPE) and average mean unsigned percentage error (AMUPE) of structural parameters of MOFs.

	Lattice Constant	LCD *^a^*	PLD *^b^*	Bond Length	Bong Angle	Torsional Angle	Unit Cell Volume	Lattice Angle	AMUPE *^c^*
MN15-L	2.09	5.95	5.62	2.46	2.74	3.17	5.71	2.38	3.76
PBE	2.81	6.43	7.29	3.05	2.18	4.85	6.62	1.35	4.32
PBEsol	1.26	3.43	4.65	1.38	1.81	2.98	1.74	2.25	2.43
PBE-D2	1.39	4.15	6.89	2.02	1.84	4.43	2.85	1.52	3.13
PBE-D3	1.34	4.03	5.56	2.24	1.33	2.76	3.33	3.52	3.01
revM06-L	0.78	3.00	3.47	1.16	1.31	1.67	1.59	0.32	1.66
revTPSS	1.83	3.62	4.19	1.79	1.93	2.47	3.86	0.40	2.51
SCAN	0.72	2.65	3.34	1.22	1.26	1.42	1.32	0.29	1.52
SOGGA	1.42	3.62	4.23	1.68	2.13	3.57	1.73	1.36	2.47
vdW-DF2	2.04	5.72	5.92	2.95	1.87	3.92	5.43	0.29	3.51

*^a^* LCD, or largest cavity diameters, is defined as the diameter of largest sphere that can fit in the pore of the MOFs. *^b^* PLD, or pore limiting diameters, is defined as the largest sphere is which able to move in a path through the MOFs. *^c^* Average mean unsigned percentage error (AMUPE) of all the structural properties.

## Supplementary Materials

The following are available online at https://www.mdpi.com/1420-3049/25/7/1552/s1. See Supplementary Materials for tables of *k* points, oxidation states, and initial magnetic moments of metal centers, magnetic ground states, experimental and calculated lattice constants, unit cell volumes, pore sizes (PLD and LCD), bond lengths, bond angles, and torsional angles of MOFs and their mean signed and unsigned errors. The supplementary materials also includes the temperatures of the X-ray measurements.
