# Supplementary material for: Improved Predictive Tools for Structural Properties of Metal–Organic Frameworks
Source: Molecules. 2020 Mar 28;25(7):1552. doi: 10.3390/molecules25071552 (PMC7180546; doi:10.3390/molecules25071552)
Supplement: Supplementary file 1 [file molecules-25-01552-s001.pdf]

SUPPLEMENTARY MATERIAL

MARCH 19, 2020

Improved predictive tools for structural properties of metal–organic frameworks

Indrani Choudhuri and Donald G. Truhlar*

Department of Chemistry, Chemical Theory Center, and Minnesota Supercomputing Institute, University of Minnesota, Minneapolis, Minnesota 55455, USA

TABLE OF CONTENTS

Table S1: Computational details and other related information of the test set of MOFs S-2

Table S2. Lattice constants (Å) of the metal–organic frameworks (MOFs) S-3

Table S3: Unit cell volumes (Å^3) of the metal–organic frameworks (MOFs) S-4

Table S4: Pore diameters (Å) of the metal–organic frameworks (MOFs) S-5

Table S5: Bond lengths (Å) of the metal–organic frameworks (MOFs) S-6

Table S6: Bond angles (deg) of the metal–organic frameworks (MOFs) S-8

Table S7: Torsional angles (deg) of the metal–organic frameworks (MOFs) S-10

Table S8: Lattice angles (deg) of the metal–organic frameworks (MOFs) S-11

Table S9: Lattice constants and band gaps of ten semiconductors calculated with revM06-L and SCAN functionals S-12

References S-13

Table S1: Computational details and other related information of the test set of MOFs

MOF[a]	Reference Code[b]	Metal Center of MOF	Oxidation State of Metal[c]	*k* points[d]	Magnetic State[e]	Initial Spin State of the Metal Ions Considered[f]	Temperatures (K) of X-ray Measurements	Reference[g]
$Ag_4C_{12}Cl_4O_8$	RORQOE	Ag	I	6×4×2	NM	--	293	1
$Cd_{12}H_{48}C_{72}N_{72}O_{48}$	GUPCUQ01	Cd	II	2×2×2	NM	--	293	2
$Cd_2H_{10}C_{16}N_4O_{10}$	PIJGEV	Cd	II	4×4×4	NM	--	295	3
$Zn_1H_4C_4O_4$	OFUWIV01	Zn	II	6×6×4	NM	--	293	4
$Li_8Zn_8H_{24}C_{72}O_{48}$	WAJJAU	Li, Zn	Li (I), Zn (II)	2×2×2	NM	--	293	5
$Co_2C_8N_{12}$	HAWVOQ01	Co	II	4×4×4	FM	3, 3	150	6
$Cu_3H_4C_{10}O_{10}$	MURCEH	Cu	II	4×4×2	AFM	−1, −1, 1, 1, −1, 1	293	7
$Cu_8H_8C_8N_{12}Cl_8$	QEJZUB01	Cu	I and II	4×4×2	AFM	1, −1, 1, −1,	90	8
$Dy_2H_{12}C_{12}N_2O_{16}$	YORSII	Dy	III	4×4×2	FM	5, 5	298	9
$Fe_4H_4C_4O_{12}$	HOGWAB	Fe	II	4×4×4	AFM	4, −4, 4, −4	283-303	10
$Fe_4P_4H_{16}C_8O_{24}$	DEMLIR	Fe	III	4×4×2	AFM	5, −5, 5, −5	298	11
$Sm_2H_{12}C_{10}O_{14}$	KOMJEC	Sm	III	4×4×4	AFM	5, −5	293	12
$Zr_{24}O_{128}C_{192}$ (UiO-66)	RUBTAK	Zr	IV	2×2×2	NM	--	298	13
$Zn_{32}O_{104}C_{192}H_{96}$ (MOF-5)	SAHYIK	Zn	II	2×2×2	NM	--	213	14

[a] Chemical formula of the unit cell

[b] The reference code associated with the structure in the Cambridge Structural Database (CSD) and CoRE MOF database.

[c] Oxidation states of the metal center according to the experimental reference

[d] *k*-points grid used for the calculations

[e] Magnetic ground state considered for the calculations

[f] Initial multiplicities of the metal ions considered for the calculations of the FM and AFM MOFs

[g] Experimental reference for the MOF

Table S2: Lattice constants (Å) of metal–organic frameworks (MOFs)

MOF	Exp.[a]	MN15–L	PBE	PBEsol	PBE–D2	PBE–D3	revM06–L	revTPSS	SCAN	SOGGA	vdW–DF2
**$Ag_4C_{12}Cl_4O_8$**	*a*=5.29	*a*=5.16	*a*=5.79	*a*=5.46	*a*=5.43	*a*=5.39	*a*=5.22	*a*=5.66	*a*=5.26	*a*=5.44	*a*=5.49
	b=6.34	*b*=6.52	*b*=6.06	*b*=6.10	*b*=6.38	*b*=6.34	*b*=6.39	*b*=5.99	*b*=6.29	*b*=6.08	*b*=6.48
	c=11.40	*c*=11.52	*c*=12.38	*c*=11.65	*c*=10.75	*c*=11.43	*c*=11.59	*c*=12.21	*c*=11.36	*c*=11.61	*c*=11.67
**$Cd_{12}H_{48}C_{72}N_{72}O_{48}$**	*a*=18.07	*a*=17.94	*a*=18.34	*a*=18.08	*a*=18.14	*a*=18.22	*a*=18.08	*a*=18.30	*a*=18.06	*a*=18.07	*a*=18.4
	b=18.07	*b*=17.94	*b*=18.34	*b*=18.08	*b*=18.14	*b*=18.22	*b*=18.08	*b*=18.30	*b*=18.06	*b*=18.07	*b*=18.4
	c=18.07	*c*=17.94	*c*=18.34	*c*=18.08	*c*=18.14	*c*=18.22	*c*=18.08	*c*=18.30	*c*=18.06	*c*=18.07	*c*=18.4
**$Cd_2H_{10}C_{16}N_4O_{10}$**	*a*=7.55	*a*=7.42	*a*=7.82	*a*=7.64	*a*=7.58	*a*=7.69	*a*=7.51	*a*=7.71	*a*=7.56	*a*=7.62	*a*=7.68
	b=7.64	*b*=7.49	*b*=7.84	*b*=7.74	*b*=7.66	*b*=7.77	*b*=7.57	*b*=7.76	*b*=7.67	*b*=7.74	*b*=7.78
	c=8.47	*c*=8.50	*c*=8.63	*c*=8.42	*c*=8.54	*c*=8.50	*c*=8.45	*c*=8.58	*c*=8.39	*c*=8.41	*c*=8.65
**$Zn_1H_4C_4O_4$**	*a*=4.83	*a*=4.83	*a*=4.96	*a*=4.87	*a*=4.87	*a*=4.90	*a*=4.74	*a*=4.94	*a*=4.83	*a*=4.87	*a*=4.90
	b=4.83	*b*=4.83	*b*=4.96	*b*=4.87	*b*=4.87	*b*=4.90	*b*=4.74	*b*=4.94	*b*=4.83	*b*=4.87	*b*=4.90
	c=6.25	*c*=6.54	*c*=6.46	*c*=6.30	*c*=6.20	*c*=6.35	*c*=6.38	*c*=6.41	*c*=6.25	*c*=6.30	*c*=6.37
**$Li_8Zn_8H_{24}C_{72}O_{48}$**	*a*=16.34	*a*=16.49	*a*=16.51	*a*=16.36	*a*=16.47	*a*=16.47	*a*=16.35	*a*=16.46	*a*=16.30	*a*=16.36	*a*=16.74
	b=16.34	*b*=16.49	*b*=16.51	*b*=16.36	*b*=16.47	*b*=16.47	*b*=16.35	*b*=16.46	*b*=16.30	*b*=16.36	*b*=16.74
	c=11.28	*c*=11.32	*c*=11.4	*c*=11.31	*c*=11.28	*c*=11.31	*c*=11.27	*c*=11.34	*c*=11.25	*c*=11.31	*c*=11.66
**$Co_2C_8N_{12}$**	*a*=5.97	*a*=5.11	*a*=6.17	*a*=5.89	*a*=5.83	*a*=5.87	*a*=5.95	*a*=5.95	*a*=5.87	*a*=6.37	*a*=5.86
	b=7.06	*b*=6.49	*b*=7.09	*b*=6.98	*b*=6.98	*b*=7.02	*b*=7.05	*b*=7.03	*b*=6.95	*b*=6.72	*b*=6.97
	c=7.41	*c*=7.59	*c*=7.35	*c*=7.29	*c*=7.39	*c*=7.39	*c*=7.36	*c*=7.31	*c*=7.26	*c*=7.29	*c*=7.23
**$Cu_3H_4C_{10}O_{10}$**	*a*=10.03	*a*=10.10	*a*=10.29	*a*=10.01	*a*=10.03	*a*=9.98	*a*=10.00	*a*=10.12	*a*=9.97	*a*=10.02	*a*=10.08
	b=5.81	*b*=5.80	*b*=6.07	*b*=5.91	*b*=5.89	*b*=5.97	*b*=5.89	*b*=5.88	*b*=5.87	*b*=5.84	*b*=5.91
	c=9.63	*c*=9.74	*c*=9.68	*c*=9.61	*c*=9.66	*c*=9.68	*c*=9.58	*c*=9.65	*c*=9.56	*c*=9.59	*c*=9.75
**$Cu_8H_8C_8N_{12}Cl_8$**	*a*=6.77	*a*=6.79	*a*=6.99	*a*=6.81	*a*=6.74	*a*=6.85	*a*=6.79	*a*=6.84	*a*=6.81	*a*=6.89	*a*=6.98
	b=6.89	*b*=6.85	*b*=7.21	*b*=6.96	*b*=6.93	*b*=6.91	*b*=6.95	*b*=7.09	*b*=6.97	*b*=6.86	*b*=7.10
	c=12.36	*c*=12.42	*c*=12.29	*c*=12.16	*c*=12.29	*c*=12.29	*c*=12.16	*c*=12.40	*c*=12.19	*c*=12.19	*c*=12.2
**$Dy_2H_{12}C_{12}N_2O_{16}$**	*a*=6.74	*a*=6.55	*a*=6.83	*a*=6.68	*a*=6.66	*a*=6.72	*a*=6.77	*a*=6.82	*a*=6.70	*a*=6.69	*a*=6.78
	b=7.81	*b*=7.78	*b*=8.02	*b*=7.90	*b*=7.78	*b*=7.89	*b*=7.77	*b*=7.92	*b*=7.78	*b*=7.89	*b*=7.91
	c=9.17	*c*=9.33	*c*=9.29	*c*=9.17	*c*=9.19	*c*=9.26	*c*=9.16	*c*=9.24	*c*=9.15	*c*=9.18	*c*=9.33
**$Fe_4H_4C_4O_{12}$**	*a*=5.93	*a*=5.64	*a*=5.78	*a*=5.68	*a*=5.87	*a*=5.87	*a*=5.92	*a*=5.68	*a*=5.92	*a*=6.26	*a*=6.19
	b=5.54	*b*=5.17	*b*=5.31	*b*=5.30	*b*=5.63	*b*=5.47	*b*=5.53	*b*=5.44	*b*=5.75	*b*=5.83	*b*=5.78
	c=7.27	*c*=7.07	*c*=7.48	*c*=7.52	*c*=7.37	*c*=7.32	*c*=7.27	*c*=7.48	*c*=7.07	*c*=7.02	*c*=7.42
**$Fe_4P_4H_{16}C_8O_{24}$**	*a*=6.61	*a*=6.29	*a*=6.77	*a*=6.74	*a*=6.83	*a*=6.78	*a*=6.52	*a*=6.71	*a*=6.63	*a*=6.68	*a*=6.66
	b=8.36	*b*=8.23	*b*=8.46	*b*=8.30	*b*=8.21	*b*=8.54	*b*=8.36	*b*=8.46	*b*=8.30	*b*=8.30	*b*=8.44
	c=9.62	*c*=9.69	*c*=9.74	*c*=9.40	*c*=9.40	*c*=9.88	*c*=9.72	*c*=9.68	*c*=9.57	*c*=9.59	*c*=9.76
**$Sm_2H_{12}C_{10}O_{14}$**	*a*=6.76	*a*=6.91	*a*=6.75	*a*=6.65	*a*=6.69	*a*=6.67	*a*=6.83	*a*=6.74	*a*=6.72	*a*=6.65	*a*=6.85
	b=7.67	*b*=7.20	*b*=8.02	*b*=7.77	*b*=7.58	*b*=7.73	*b*=7.35	*b*=7.91	*b*=7.53	*b*=7.77	*b*=7.58
	c=8.05	*c*=7.89	*c*=8.19	*c*=8.04	*c*=7.90	*c*=8.12	*c*=7.99	*c*=8.19	*c*=8.00	*c*=8.03	*c*=8.19
**UiO-66**	*a*=*b*=*c*= 20.70	*a*=*b*=*c*= 20.61	*a*=*b*=*c*= 21.12	*a*=*b*=*c*= 20.96	*a*=*b*=*c*= 21.08	*a*=*b*=*c*= 21.07	*a*=*b*=*c*= 20.57	*a*=*b*=*c*= 21.11	*a*=*b*=*c*= 20.63	*a*=*b*=*c*= 20.95	*a*=*b*=*c*= 20.16
**MOF-5**	*a*=*b*=*c*= 25.66	*a*=*b*=*c*= 25.86	*a*=*b*=*c*= 26.09	*a*=*b*=*c*= 25.88	*a*=*b*=*c*= 25.07	*a*=*b*=*c*= 26.06	*a*=*b*=*c*= 25.79	*a*=*b*=*c*= 26.01	*a*=*b*=*c*= 25.83	*a*=*b*=*c*= 25.86	*a*=*b*=*c*= 26.19
**MSE**		**-0.05**	**0.20**	**-0.03**	**0.04**	**0.09**	**-0.02**	**0.13**	**-0.02**	**0.04**	**0.19**
**MUE**		**0.16**	**0.26**	**0.11**	**0.15**	**0.14**	**0.07**	**0.18**	**0.06**	**0.12**	**0.22**
**MUPA (%)**		**2.09**	**2.81**	**1.26**	**1.39**	**1.34**	**0.78**	**1.83**	**0.72**	**1.42**	**2.04**

[a] The references for the experimental lattice constants are given in table S1.

Table S3: Unit cell volumes (Å^3) of the metal–organic frameworks (MOFs)

MOF	Exp.[a]	MN15-L	PBE	PBEsol	PBE–D2	PBE–D3	revM06 –L	revTPSS	SCAN	SOGGA	vdW –DF2
$Ag_4C_{12}Cl_4O_8$	382.09	387.73	427.34	386.54	372.05	390.65	386.54	409.06	375.79	383.00	415.59
$Cd_{12}H_{48}C_{72}N_{72}O_{48}$	5907.36	5771.61	6167.81	5914.82	5968.28	6045.56	5910.69	6127.55	5892.93	5900.64	6232.68
$Cd_2H_{10}C_{16}N_4O_{10}$	435.56	431.81	464.86	437.40	434.76	445.74	431.93	453.66	430.46	435.95	457.62
$Zn_1H_4C_4O_4$	134.58	119.55	147.97	137.48	135.19	141.69	126.46	142.13	134.29	137.34	138.13
$Li_8Zn_8H_{24}C_{72}O_{48}$	3011.16	3080.00	3107.53	3027.04	3057.86	3067.34	3012.63	3072.81	2988.82	3027.40	3272.48
$Co_2C_8N_{12}$	312.15	532.18	334.29	299.48	284.90	288.37	308.76	305.49	296.18	312.15	295.51
$Cu_3H_4C_{10}O_{10}$	552.22	560.24	592.33	559.34	575.42	567.42	540.48	565.84	549.60	562.22	582.48
$Cu_8H_8C_8N_{12}Cl_8$	576.57	578.06	619.38	575.69	573.49	582.02	573.95	601.84	579.22	570.57	604.48
$Dy_2H_{12}C_{12}N_2O_{16}$	448.06	423.43	477.26	451.41	433.57	450.79	442.18	465.79	439.80	452.50	459.15
$Fe_4H_4C_4O_{12}$	238.62	206.60	229.25	224.78	243.18	235.19	238.69	231.17	238.62	245.38	266.13
$Fe_4P_4H_{16}C_8O_{24}$	531.61	502.13	558.15	526.17	527.66	572.86	529.45	549.75	526.61	539.61	548.27
$Sm_2H_{12}C_{10}O_{14}$	384.47	364.78	409.14	382.35	367.92	388.02	370.66	400.90	373.01	356.55	394.03
**UiO-66**	8870.26	8756.44	9429.46	9218.97	9371.18	9353.97	8714.89	9408.56	8802.82	9200.85	9407.48
**MOF-5**	16913.2	17311.9	17769.4	17337	17717.2	17703.6	17171.7	17603.01	17249.9	17294.9	17602
**MSE**		-19.69	145.44	55.75	-97.48	109.66	-1.67	-34.30	12.86	51.08	-173.85
**MUE**		33.69	146.78	6.50	108.36	113.55	8.22	36.65	35.61	56.89	173.85
**MUPE (%)**		**5.71**	**6.62**	**1.74**	**2.85**	**3.33**	**1.59**	**3.86**	**1.32**	**1.73**	**5.43**

[a] The experimental unit cell volume is calculated using the zeo++ software.[15,16,17]

Table S4: Pore diameters (Å) of the metal–organic frameworks (MOFs)

MOF	Acc.[a]		MN15-L		PBE		PBEsol		PBE–D2		PBE–D3		revM06-L		revTPSS		SCAN		SOGGA		vdW-DF2	
	LCD	PLD	LCD	PLD	LCD	PLD	LCD	PLD	LCD	PLD	LCD	PLD	LCD	PLD	LCD	PLD	LCD	PLD	LCD	PLD	LCD	PLD
$Ag_4C_{12}Cl_4O_8$	1.36	1.30	1.48	1.47	1.65	1.65	1.47	1.47	1.32	1.32	1.37	1.31	1.32	1.26	1.53	1.53	1.32	1.32	1.45	1.45	1.48	1.35
$Cd_{12}H_{48}C_{72}N_{72}O_{48}$	12.58	12.58	12.43	12.43	12.72	12.72	12.49	12.49	12.53	12.53	12.60	12.60	12.57	12.57	12.70	12.70	12.45	12.45	12.48	12.48	12.72	12.72
$Cd_2H_{10}C_{16}N_4O_{10}$	2.02	2.02	1.97	1.95	2.07	2.06	2.03	2.03	1.99	1.97	2.05	2.02	2.01	2.01	2.09	2.09	2.01	2.01	2.02	2.02	2.13	2.13
$Zn_1H_4C_4O_4$	1.70	1.70	1.74	1.74	1.85	1.85	1.73	1.73	1.63	1.63	1.72	1.72	1.61	1.61	1.82	1.82	1.63	1.63	1.73	1.73	1.51	1.51
$Li_8Zn_8H_{24}C_{72}O_{48}$	7.48	7.48	7.5	7.5	7.52	7.52	7.43	7.43	7.50	7.49	7.50	7.49	7.43	7.43	7.47	7.47	7.39	7.39	7.43	7.43	7.66	7.66
$Co_2C_8N_{12}$	1.85	1.85	1.71	1.71	2.02	2.02	1.80	1.80	1.67	1.67	1.63	1.57	1.84	1.84	1.82	1.82	1.77	1.77	1.80	1.80	1.80	1.67
$Cu_3H_4C_{10}O_{10}$	1.80	1.80	1.7	1.7	1.93	1.93	1.70	1.62	1.75	1.75	1.72	1.72	1.65	1.65	1.76	1.76	1.70	1.70	1.70	1.70	1.77	1.70
$Cu_8H_8C_8N_{12}Cl_8$	1.37	1.29	1.35	1.26	1.53	1.51	1.41	1.37	1.38	1.18	1.43	1.29	1.39	1.33	1.48	1.45	1.42	1.34	1.41	1.34	1.49	1.27
$Dy_2H_{12}C_{12}N_2O_{16}$	1.81	1.81	1.66	1.65	1.82	1.77	1.69	1.69	1.54	1.54	1.56	1.66	1.75	1.70	1.79	1.79	1.65	1.63	1.71	1.71	1.65	1.65
$Fe_4H_4C_4O_{12}$	1.21	1.21	1.05	1.02	1.07	1.01	1.08	1.05	1.07	1.03	1.20	1.54	1.13	1.11	1.23	1.20	1.23	1.23	1.41	1.40	1.37	1.36
$Fe_4P_4H_{16}C_8O_{24}$	1.37	1.35	1.24	1.24	1.46	1.36	1.39	1.38	1.37	1.25	1.49	1.55	1.31	1.29	1.44	1.31	1.37	1.23	1.35	1.28	1.41	1.27
$Sm_2H_{12}C_{10}O_{14}$	1.36	1.30	1.5	1.74	1.93	1.91	1.92	1.92	1.86	1.50	1.96	1.90	1.81	1.78	1.95	1.95	1.92	1.92	1.92	1.90	1.99	1.99
UiO-66	8.88	3.79	8.82	3.76	9.37	4.05	9.26	3.99	9.34	4.03	9.09	3.92	8.80	3.74	9.12	3.93	8.75	3.71	9.26	3.98	9.39	4.06
MOF-5	14.94	7.84	15.08	7.92	15.24	8.03	15.10	7.93	15.23	8.02	15.23	8.02	15.03	7.89	15.18	7.99	15.06	7.92	15.08	7.92	15.32	8.08
MSE			-0.07	-0.06	0.13	0.10	0.02	-0.001	-0.01	-0.07	-0.01	0.03	-0.05	-0.05	0.07	0.06	-0.04	-0.04	0.03	0.02	0.10	0.04
MUE			**0.12**	**0.10**	**0.15**	**0.13**	**0.09**	**0.09**	**0.12**	**0.14**	**0.10**	**0.10**	**0.06**	**0.06**	**0.09**	**0.08**	**0.07**	**0.08**	**0.09**	**0.08**	**0.16**	**0.14**
MUPE (%)			**5.95**	**5.62**	**6.43**	**7.29**	**3.43**	**4.65**	**4.15**	**6.89**	**4.03**	**5.56**	**3.00**	**3.47**	**3.62**	**4.19**	**2.65**	**3.34**	**3.62**	**4.23**	**5.72**	**5.92**

[a] The accurate unit cell volume is calculated using the zeo++ software.[15,16,17]

Table S5: Bond lengths (Å) of the metal–organic frameworks (MOFs)

MOF	Bond length type	Exp.[a]	MN15–L	PBE	PBEsol	PBE–D2	PBE–D3	revM06–L	revTPSS	SCAN	SOGGA	vdW–DF2
$Ag_4C_{12}Cl_4O_8$	Ag-O	2.375	2.500	2.441	2.327	2.504	2.425	2.459	2.341	2.388	2.298	2.469
		2.468	2.522	2.38	2.41	2.451	2.474	2.496	2.502	2.471	2.444	2.527
		2.442	2.494	2.543	2.393	2.359	2.380	2.421	2.383	2.369	2.319	2.436
		2.461	2.519	2.565	2.453	2.452	2.457	2.462	2.407	2.408	2.386	2.469
	Ag-Cl	2.815	2.952	2.907	2.811	2.75	2.829	2.883	2.878	2.811	2.800	2.893
$Cd_{12}H_{48}C_{72}N_{72}O_{48}$	Cd-N	2.246	2.266	2.298	2.256	2.266	2.279	2.25	2.289	2.265	2.252	2.297
$Cd_2H_{10}C_{16}N_4O_{10}$	Cd-N	2.406	2.428	2.421	2.378	2.421	2.412	2.43	2.417	2.388	2.371	2.428
		2.511	2.573	2.56	2.49	2.56	2.543	2.541	2.521	2.504	2.479	2.548
	Cd-O	2.332	2.396	2.373	2.358	2.373	2.358	2.372	2.359	2.354	2.353	2.395
		2.321	2.328	2.395	2.356	2.395	2.376	2.324	2.363	2.333	2.352	2.37
		2.527	2.635	2.641	2.543	2.652	2.584	2.571	2.582	2.52	2.542	2.57
		2.299	2.403	2.332	2.285	2.332	2.31	2.355	2.335	2.305	2.281	2.355
		2.289	2.291	2.381	2.329	2.382	2.349	2.287	2.334	2.31	2.328	2.339
$Zn_1H_4C_4O_4$	Zn-O	1.943	1.984	1.981	1.952	1.973	1.974	1.937	1.977	1.944	1.95	1.981
		1.947	1.977	1.983	1.952	1.965	2.017	1.935	1.983	1.946	1.951	1.995
$Li_8Zn_8H_{24}C_{72}O_{48}$	Li-O	1.866	1.876	1.886	1.87	1.879	1.878	1.891	1.874	1.867	1.87	1.895
		2.001	2.02	2.03	1.993	1.999	2.02	2.07	2.001	1.988	1.993	2.048
		2.018	1.925	2.035	2.0152	2.088	2.039	2.076	2.016	1.995	2.015	2.053
		1.905	2.023	1.918	1.897	1.895	2.003	1.948	1.921	2.001	1.897	1.998
	Zn-O	1.900	1.936	1.932	1.907	1.926	1.926	1.922	1.936	1.901	1.907	1.943
$Co_2C_8N_{12}$	Co-N	2.096	2.013	2.048	2.017	1.940	1.909	2.084	2.030	2.011	1.992	1.909
		2.153	2.113	2.315	2.128	2.148	2.147	2.165	2.160	2.121	2.115	2.322
$Cu_3H_4C_{10}O_{10}$	Cu-O	1.951	1.982	1.99	1.938	1.965	1.969	1.945	1.949	1.928	1.923	1.981
		1.978	2.005	2.027	1.985	2.002	2.000	1.976	1.987	1.96	1.981	2.022
		1.956	1.989	2.05	1.974	1.983	1.985	1.966	1.954	1.942	1.951	2.011
		1.937	1.987	2.165	1.936	1.951	1.949	1.942	1.940	1.918	1.905	1.959
		2.103	2.089	2.141	2.096	2.127	2.154	2.124	2.110	2.094	2.0816	2.158
$Cu_8H_8C_8N_{12}Cl_8$	Cu-Cl	2.409	2.466	2.468	2.403	2.435	2.447	2.442	2.430	2.421	2.416	2.504
	Cu-N	1.937	1.964	2.165	1.919	1.941	1.943	1.925	1.960	1.924	1.918	1.987

continued on next page

MOF	Bond length type	Exp.[a]	MN15–L	PBE	PBEsol	PBE–D2	PBE–D3	revM06–L	revTPSS	SCAN	SOGGA	vdW–DF2
**$Dy_2H_{12}C_{12}N_2O_{16}$**	**Dy-O**	2.377	2.357	2.385	2.359	2.359	2.374	2.352	2.366	2.344	2.341	2.456
		2.405	2.423	2.418	2.359	2.370	2.386	2.412	2.421	2.382	2.363	2.499
		2.289	2.281	2.308	2.274	2.278	2.305	2.287	2.298	2.275	2.272	2.313
		2.336	2.429	2.363	2.32	2.331	2.297	2.356	2.343	2.324	2.315	2.367
		2.266	2.208	2.349	2.275	2.258	2.278	2.233	2.287	2.256	2.275	2.28
		2.388	2.385	2.432	2.401	2.408	2.449	2.4	2.407	2.387	2.391	2.425
		2.423	2.411	2.506	2.476	2.542	2.506	2.437	2.465	2.457	2.478	2.491
		2.398	2.503	2.433	2.401	2.45	2.444	2.422	2.420	2.411	2.394	2.449
**$Fe_4H_4C_4O_{12}$**	**Fe-O**	2.060	1.987	2.113	2.118	2.097	2.121	2.018	2.099	1.996	2.006	2.129
		2.091	2.005	2.055	2.081	2.07	2.189	2.061	2.150	2.029	2.002	2.129
		2.157	2.027	2.09	2.042	2.057	2.033	2.169	2.077	2.209	2.25	2.234
		2.145	1.915	2.064	2.021	2.051	2.088	2.209	2.031	2.094	1.96	2.03
		2.194	2.211	2.288	2.248	2.328	2.218	2.247	2.160	2.264	2.25	2.138
		2.265	2.105	2.23	2.166	2.238	2.194	2.271	2.102	2.264	2.165	2.042
**$Fe_4P_4H_{16}C_8O_{24}$**	**Fe-O**	2.103	1.978	1.987	1.999	1.932	1.929	2.021	1.933	2.016	2.003	1.938
		1.941	1.938	1.968	1.945	1.961	1.96	1.938	1.965	1.94	1.93	1.987
		1.919	1.967	2.002	1.967	1.974	1.99	1.962	1.988	1.969	1.976	1.993
		2.008	1.985	2.04	2.068	2.066	2.096	2.011	2.026	2.009	2.000	2.098
		1.961	2.085	1.997	1.991	1.997	2.062	1.937	2.130	2.037	2.086	2.148
		2.103	2.089	2.141	2.096	2.127	2.154	2.124	2.110	2.094	2.0816	2.158
**$Sm_2H_{12}C_{10}O_{14}$**	**Sm-O**	2.402	2.384	2.294	2.28	2.474	2.294	2.356	2.294	2.306	2.278	2.3235
		2.423	2.411	2.513	2.398	2.372	2.409	2.426	2.417	2.412	2.394	2.49
		2.440	2.489	2.97	2.405	2.391	2.654	2.468	2.447	2.417	2.402	2.469
		2.403	2.534	2.138	2.378	2.29	2.406	2.465	2.398	2.407	2.373	2.445
		2.309	2.408	2.406	2.398	2.343	2.417	2.35	2.426	2.396	2.3987	2.422
		2.504	2.535	2.55	2.524	2.515	2.545	2.555	2.541	2.536	2.515	2.571
		2.549	2.494	2.589	2.489	2.546	2.52	2.4953	2.498	2.476	2.483	2.439
		2.456	2.458	2.501	2.472	2.372	2.478	2.462	2.474	2.455	2.469	2.474
**UiO-66**	**Zr-O**	2.23	2.22	**2.26**	**2.23**	**2.25**	**2.27**	2.22	2.27	2.22	2.22	2.26
		2.10	2.09	**2.13**	**2.12**	**2.13**	**2.14**	2.09	2.14	2.11	2.11	2.15
**MOF-5**	**Zn-O**	1.91	1.92	**1.97**	**1.94**	**1.96**	**1.96**	1.92	1.96	1.93	1.93	1.98
**MSE (Å)**			**0.014**	**0.04**	**-0.008**	**0.008**	**0.02**	**0.012**	**0.008**	**-0.005**	**-0.015**	**0.03**
**MUE (Å)**			**0.054**	**0.069**	**0.031**	**0.045**	**0.049**	**0.026**	**0.039**	**0.027**	**0.037**	**0.064**
**MUPE (%)**			**2.46**	**3.05**	**1.38**	**2.02**	**2.24**	**1.16**	**1.79**	**1.22**	**1.68**	**2.95**

[a] The references for the experimental bond lengths are given in Table S1.

Table S6: Bond angles (deg) of the metal–organic frameworks (MOFs) calculated using various functionals.

MOF	Bond angle type	Exp.[a]	MN15–L	PBE	PBEsol	PBE–D2	PBE–D3	revM06–L	revTPSS	SCAN	SOGGA	vdW–DF2
**$Ag_4C_{12}Cl_4O_8$**		65.96	64.73	65.57	66.82	66.46	65.93	64.91	66.14	66.21	67.02	64.4
	**O-Ag-O**	74.16	71.33	75.24	73.01	77.00	74.71	73.27	72.90	72.76	72.74	75.09
		108.82	110.25	110.64	117.90	98.41	110.53	112.51	122.49	112.35	117.94	110.85
		110.23	116.55	103.76	107.67	109.21	111.42	111.84	103.61	110.46	107.70	110.98
		137.82	132.08	135.44	134.00	131.55	136.66	164.62	122.49	135.33	133.91	137.05
		139.57	145.16	145.14	143.14	143.13	139.42	142.29	143.42	140.89	142.94	142.01
	**O-Ag-Cl**	74.94	78.09	74.32	72.69	77.63	75.29	75.57	73.16	73.58	72.60	75.63
		68.37	66.26	79.13	70.40	68.73	68.80	67.10	69.21	69.23	70.67	66.12
		137.90	139.41	132.12	133.66	142.81	139.5	136.15	131.89	136.31	133.73	134.76
		135.63	132.56	130.76	127.39	136.71	135.18	132.99	125.63	134.30	127.48	136.79
**$Cd_{12}H_{48}C_{72}N_{72}O_{48}$**	**N-Cd-N**	131.10	137.36	133.00	133.00	133.18	133.12	131.57	132.75	133.98	132.96	134
		99.85	97.59	99.14	99.14	99.08	99.10	99.68	99.23	98.78	99.16	98.78
**$Cd_2H_{10}C_{16}N_4O_{10}$**	**N-Cd-N**	66.38	65.96	66.19	67.55	66.19	66.63	65.52	66.43	66.81	67.77	66.46
	**N-Cd-O**	74.73	77.54	73.80	74.21	73.80	74.57	76.08	74.01	75.13	74.22	75.5
	**N-Cd-O**	82.18	83.29	83.79	82.01	75.06	82.47	82.35	82.60	81.91	82.00	81.68
		97.58	89.95	101.34	100.53	101.34	99.87	94.71	99.28	98.67	100.51	97.68
		75.99	75.13	75.06	75.41	75.06	75.62	75.86	75.72	75.95	75.44	75.82
	**O-Cd-O**	84.29	81.48	83.95	84.91	83.95	85.23	82.19	83.37	83.99	84.78	83.98
		85.42	86.93	83.20	83.32	83.20	84.34	86.35	83.27	84.10	83.15	85.17
		84.52	89.27	81.77	81.39	81.77	81.73	85.69	83.45	83.24	81.43	83.35
**$Zn_1H_4C_4O_4$**		102.9	105.55	101.07	101.85	102.87	103.11	105.18	100.70	101.70	101.82	105.54
	**O-Zn-O**	114.89	112.23	112.49	112.83	115.51	115.82	113.93	112.38	114.63	112.83	115.92
		107.1	107.01	107.33	106.45	109.49	108.76	108.53	107.08	109.38	106.44	107.33
**$Li_8Zn_8H_{24}C_{72}O_{48}$**		105.55	103.90	106.68	105.25	106.70	105.54	105.52	105.13	104.61	105.25	105.44
	**O-Li-O**	84.15	84.65	84.35	84.14	83.53	83.92	84.84	84.17	84.44	84.14	84.99
		137.90	139.14	137.12	137.66	136.41	138.40	138.21	135.83	137.97	137.66	138.64
		107.70	107.98	109.19	108.78	111.00	109.39	107.69	108.35	108.20	108.78	108.93
		94.12	93.25	93.29	93.30	94.76	93.89	93.34	91.91	93.18	93.30	95.58
	**O-Zn-O**	116.94	117.66	116.95	117.53	115.88	116.79	116.92	117.71	117.92	117.53	116.02
		121.28	121.28	121.29	121.10	121.47	121.29	122.07	120.90	121.00	121.10	122.42
	**Li-O-Zn**	94.09	93.87	93.91	94.31	95.10	94.52	93.67	94.52	93.86	94.31	94.35
		95.20	95.80	95.52	95.43	93.99	95.58	95.32	96.03	95.52	95.43	96.59

continued in next page

MOF	Bond angle type	Exp.[a]	MN15−L	PBE	PBEsol	PBE−D2	PBE−D3	revM06−L	revTPSS	SCAN	SOGGA[illegible]	vdW−DF2
**$Co_2C_8N_{12}$**	**N-Co-N**	88.95	88.71	89.28	88.99	89.02	89.03	88.96	88.99	88.99	89.47	89.12
		85.14	86.29	87.26	85.48	85.68	86.02	84.95	91.00	85.10	85.53	86.64
		91.05	91.29	90.71	91.00	90.97	90.97	91.04	91.09	91.00	90.53	90.87
		94.86	93.58	93.75	94.51	93.99	94.17	94.55	93.66	94.11	94.58	93.35
**$Cu_3H_4C_{10}O_{10}$**	**O-Cu-O**	94.86	93.58	93.75	94.51	93.99	94.17	94.55	93.66	94.11	94.58	93.35
		95.57	97.28	95.05	96.12	95.50	95.48	95.91	96.55	96.26	94.76	96.9
		95.48	95.27	98.10	96.56	96.38	96.95	95.70	96.48	96.35	96.46	98.97
		82.83	81.81	79.95	81.33	82.02	82.29	82.71	81.18	81.45	83.04	80.04
		89.53	89.21	90.56	89.21	89.78	89.20	89.20	89.44	90.19	89.58	89.31
**$Cu_8H_8C_8N_{12}Cl_8$**	**N-Cu-Cl**	89.32	88.29	90.38	89.84	88.52	89.52	89.50	89.72	89.57	91.46	89.4
		90.68	91.70	89.61	90.15	91.47	90.47	90.49	90.27	90.42	88.53	90.59
**$Dy_2H_{12}C_{12}N_2O_{16}$**	**O-Dy-O**	75.84	80.49	78.84	79.90	82.84	79.84	78.03	78.12	79.06	80.17	79.87
		75.51	75.05	77.85	78.05	75.90	76.75	76.19	77.21	76.69	78.13	76.29
		73.54	68.04	76.14	75.20	69.36	72.34	73.00	74.74	73.22	75.01	71.69
		74.21	77.77	71.81	71.08	71.76	72.44	73.78	71.96	72.49	70.64	73.4
		80.01	86.18	75.70	76.55	82.74	78.72	79.45	76.65	79.23	76.68	80.64
		85.78	82.72	84.41	85.04	87.51	84.91	84.89	84.33	85.70	84.91	84.42
		83.15	77.30	87.43	86.96	82.89	84.78	82.22	85.47	83.69	86.74	83.42
		76.09	75.61	76.79	76.30	76.93	76.71	75.60	77.44	76.33	76.04	74.98
**$Fe_4H_4C_4O_{12}$**	**O-Fe-O**	88.31	93.68	85.09	84.70	83.16	85.52	88.11	86.45	91.95	84.006	84.79
		73.86	79.31	71.75	73.86	71.51	74.61	72.91	77.18	72.98	82.002	81.89
		85.59	84.94	83.73	83.14	82.64	83.60	84.69	84.79	82.21	73.28	79.51
		104.87	98.64	98.73	100.22	98.16	107.91	106.67	106.62	103.09	106.96	102.13
		87.34	89.35	90.41	89.32	88.82	88.94	86.20	89.18	85.264	82.25	84.76
		93.86	90.08	92.34	92.33	92.03	92.58	89.31	91.91	84.15	91.73	91.59
**$Fe_4P_4H_{16}C_8O_{24}$**	**O-Fe-O**	94.16	92.87	94.21	94.61	94.48	94.72	93.24	93.87	94.23	94.06	94.43
		81.65	81.32	81.07	82.41	82.59	81.50	80.78	81.28	81.54	81.97	81.19
		84.39	86.46	83.12	87.93	87.34	83.97	84.22	83.32	84.65	83.99	82.98
		90.43	92.72	89.23	90.89	91.01	89.96	89.62	89.71	90.54	89.28	93.18
		93.43	91.09	94.70	92.50	91.77	93.38	94.53	94.19	93.40	94.29	91.85
		90.84	85.37	92.39	90.33	89.99	90.53	90.66	91.82	90.08	90.89	89.5
		78.47	81.75	76.3	73.29	74.87	74.84	78.54	73.88	79.88	72.95	83.68
		152.01	154.95	151.92	151.05	154.89	148.84	151.37	151.03	151.07	149.11	157.07
		161.34	164.49	164.71	162.51	165.83	158.57	161.26	158.07	164.96	163.21	165.53
		174.05	175.6	174.99	171.36	178.03	168.62	172.36	171.73	172.03	174.46	168.95
		87.09	87.33	87.52	82.09	82.05	84.6	85.72	88.40	85.61	83.09	82.99
		88.92	92.21	87.9	89.92	86.58	91.21	90.35	92.88	90.32	89.88	96.39
		120.59	119.68	123.46	123.57	123.79	122.9	121.29	119.19	124.08	123.17	125.68
**$Sm_2H_{12}C_{10}O_{14}$**	**O-Sm-O**	51.02	51.96	51.32	51.88	51.34	51.49	51.00	51.79	51.68	52.03	51.34
		75.42	76.97	74.56	73.86	74.16	74.19	76.63	73.90	74.94	73.82	74.24
		77.21	69.06	74.54	75.28	77.18	75.80	70.28	74.42	77.40	75.35	78.25
		75.97	65.62	74.06	75.56	77.42	75.33	69.86	75.10	76.87	75.50	76.93
		74.86	79.17	77.73	75.75	73.69	77.23	78.50	76.19	73.61	75.61	76.93
		67.35	71.30	65.91	66.30	69.55	66.70	68.71	66.08	66.83	66.10	68.83
**UiO-66**	**Zr-O-Zr**	112.76	112.76	110.40	110.21	110.08	112.76	112.8	112.76	116.50	110.04	110.69
	**O-Zr-O**	77.30	77.30	76.09	76.02	75.92	71.03	81.38	77.30	81.69	75.91	76.41
**MOF-5**	**Zn-O-Zn**	109.47	109.47	109.47	109.47	109.47	109.47	109.55	109.47	109.47	109.47	109.47
	**O-Zn-O**	106.36	106.36	107.34	107	107.35	107.40	108.47	107.09	106.87	106.93	107.59
**MSE (°)**			**0.10**	**0.06**	**-0.34**	**-0.11**	**-0.05**	**-0.02**	**-0.43**	**0.01**	**-0.42**	0.26
**MUE (°)**			**2.46**	**2.00**	**1.71**	**1.75**	**1.15**	**1.20**	**1.94**	**1.19**	**1.95**	1.77
**MUPE (%)**			**2.74**	**2.18**	**1.81**	**1.84**	**1.33**	**1.31**	**1.93**	**1.26**	**2.13**	**1.87**

[a] The references for the experimental bond angles are given in Table S1.

Table S7: Torsional angles (deg) of the metal–organic frameworks (MOFs) calculated using various local and meta functionals.

MOF	Torsional angle type	Exp.[a]	MN15-L	PBE	PBEsol	PBE–D2	PBE–D3	revM06-L	revTPSS	SCAN	SOGGA	vdW-DF2
$Ag_4C_{12}Cl_4O_8$	Ag-O-Ag-Cl	142.58	134.67	132.45	125.89	158.72	135.42	135.01	122.88	137.23	125.78	139.92
$Cd_{12}H_{48}C_{72}N_{72}O_{48}$	N-Cd-N-C	140.60	141.83	145.95	142.96	142.99	142.99	141.05	143.43	143.47	143.06	143.55
$Cd_2H_{10}C_{16}N_4O_{10}$	O-Cd-N-C	123.80	123.43	127.59	124.03	124.03	124.37	123.73	124.06	124.09	123.92	126.52
$Zn_1H_4C_4O_4$	O-Zn-O-C	151.50	169.98	144.71	144.12	145.99	150.26	146.89	143.42	150.02	144.12	157.51
$Li_8Zn_8H_{24}C_{72}O_{48}$	Li-O-Zn-O	128.40	127.80	130.29	127.96	131.39	130.04	128.33	125.71	127.73	127.96	133.44
$Co_2C_8N_{12}$	N-Co-N-C	150.30	166.86	164.66	148.88	154.73	154.11	150.42	150.76	149.66	171.06	161.48
$Cu_3H_4C_{10}O_{10}$	Cu-O-Cu-O	117.64	116.81	129.2	119.02	118.91	123.86	117.28	119.54	122.23	118.90	121.03
$Cu_8H_8C_8N_{12}Cl_8$	Cu-Cl-Cu-Cl	153.40	149.82	157.94	152.41	153.85	152.65	151.65	152.69	153.17	148.36	141.86
$Dy_2H_{12}C_{12}N_2O_{16}$	O-Dy-O-C	165.23	173.83	174.61	167.66	173.46	169.97	168.08	168.05	169.91	167.79	171.28
$Fe_4H_4C_4O_{12}$	O-Fe-O-C	164.70	168.11	162.02	170.05	163.33	169.84	159.21	164.91	165.53	158.15	171.83
$Fe_4P_4H_{16}C_8O_{24}$	Fe-O-P-O	93.40	93.48	101.52	102.58	120.05	105.31	89.73	94.37	94.19	95.03	102.22
$Sm_2H_{12}C_{10}O_{14}$	O-Sm-O-C	131.55	126.77	141.87	136.30	134.43	134.57	127.74	136.71	132.35	136.34	136.67
**UiO-66**	Zr-O-Zr-O	154.96	154.96	157.87	158.09	158.21	154.96	156.96	154.96	150.11	158.26	157.66
**MOF-5**	Zn-O-Zn-O	120	120	120	120	120	120	120	120	120	120	120
**MSE (°)**			**2.16**	**3.75**	**0.13**	**4.43**	**2.16**	**-1.57**	**-0.96**	**0.11**	**-0.05**	**3.35**
**MUE (°)**			**4.74**	**6.55**	**3.98**	**5.41**	**3.47**	**2.34**	**3.48**	**2.00**	**5.22**	**5.37**
**MUPE (%)**			**3.17**	**4.85**	**2.98**	**4.43**	**2.76**	**1.67**	**2.47**	**1.42**	**3.57**	**3.92**

[a] The references for the experimental bond lengths are given in Table S1.

Table S8: Lattice angles (deg) of the metal–organic frameworks (MOFs) calculated using various local and meta functionals.

MOF	Exp.[a]	MN15–L	PBE	PBEsol	PBE–D2	PBE–D3	revM06–L	revTPSS	SCAN	SOGGA	vdW–DF2
$Ag_4C_{12}Cl_4O_8$	α=90	α=90	α=90	α=90	α=90	α=90	α=90	α=90	α=90	α=90	α=90
	β=90	β=86.73	β=97.92	β=94.99	β=89.59	β=91.30	β=90.97	β=96.02	β=91.33	β=95.19	β=91.53
	γ=90	γ=90	γ=90	γ=90	γ=90	γ=90	γ=90	γ=90	γ=90	γ=90	γ=90
$Cd_{12}H_{48}C_{72}N_{72}O_{48}$	α=90	α=90	α=90	α=90	α=90	α=90	α=90	α=90	α=90	α=90	α=90
	β=90	β=90	β=90	β=90	β=90	β=90	β=90	β=90	β=90	β=90	β=90
	γ=90	γ=90	γ=90	γ=90	γ=90	γ=90	γ=90	γ=90	γ=90	γ=90	γ=90
$Cd_2H_{10}C_{16}N_4O_{10}$	α=71.4	α=72.6	α=70.18	α=70.40	α=69.73	α=70.06	α=71.98	α=71.00	α=70.94	α=70.39	α=70.53
	β=73	β=76	β=71.85	β=71.97	β=72.57	β=72.37	β=74.20	β=72.26	β=72.75	β=71.96	β=73.15
	γ=74.9	γ=77.14	γ=74.36	γ=73.73	γ=73.81	γ=73.77	γ=75.19	γ=74.49	γ=74.24	γ=73.74	γ=74.27
$Zn_1H_4C_4O_4$	α=90	α=90	α=90	α=90	α=90	α=90	α=90	α=90	α=90	α=90	α=90
	β=108.5	β=110.65	β=114.42	β=104.64	β=105.35	β=105.83	β=104.28	β=110.92	β=105.23	β=104.62	β=104.52
	γ=90	γ=90	γ=90	γ=90	γ=90	γ=90	γ=90	γ=90	γ=90	γ=90	γ=90
$Li_8Zn_8H_{24}C_{72}O_{48}$	α=90	α=90	α=90	α=90	α=90	α=90	α=90	α=90	α=90	α=90	α=90
	β=90	β=90	β=90	β=90	β=90	β=90	β=90	β=90	β=90	β=90	β=90
	γ=90	γ=90	γ=90	γ=90	γ=90	γ=90	γ=90	γ=90	γ=90	γ=90	γ=90
UiO-66	α=90	α=90	α=90	α=90	α=90	α=90	α=90	α=90	α=90	α=90	α=90
	β=90	β=90	β=90	β=90	β=90	β=90	β=90	β=90	β=90	β=90	β=90
	γ=90	γ=90	γ=90	γ=90	γ=90	γ=90	γ=90	γ=90	γ=90	γ=90	γ=90
MOF-5	α=90	α=90	α=90	α=90	α=90	α=90	α=90	α=90	α=90	α=90	α=90
	β=90	β=90	β=90	β=90	β=90	β=90	β=90	β=90	β=90	β=90	β=90
	γ=90	γ=90	γ=90	γ=90	γ=90	γ=90	γ=90	γ=90	γ=90	γ=90	γ=90
$Co_2C_8N_{12}$	α=90	α=90	α=90	α=90	α=90	α=90	α=90	α=90	α=90	α=90	α=90
	β=90	β=90	β=90	β=90	β=90	β=90	β=90	β=90	β=90	β=90	β=90
	γ=90	γ=90	γ=90	γ=90	γ=90	γ=90	γ=90	γ=90	γ=90	γ=90	γ=90
$Cu_3H_4C_{10}O_{10}$	α=98.6	α=100.43	α=96.91	α=98.99	α=99.17	α=98.17	α=99.44	α=98.48	α=99.00	α=108.78	α=98.91
	β=92	β=91.99	β=91.40	β=92.02	β=92.04	β=91.74	β=92.32	β=91.85	β=92.02	β=97.90	β=91.86
	γ=94.3	γ=93.61	γ=96.98	γ=94.95	γ=95.39	γ=95.47	γ=94.39	γ=95.00	γ=95.62	γ=90.93	γ=94.87
$Cu_8H_8C_8N_{12}Cl_8$	α=90	α=90	α=90	α=90	α=90	α=90	α=90	α=90	α=90	α=90	α=90
	β=90	β=90	β=90	β=90	β=90	β=90	β=90	β=90	β=90	β=90	β=90
	γ=90	γ=90	γ=90	γ=90	γ=90	γ=90	γ=90	γ=90	γ=90	γ=90	γ=90
$Dy_2H_{12}C_{12}N_2O_{16}$	α=95.6	α=96.49	α=90	α=96.12	α=95.13	α=95.89	α=95.60	α=96.07	α=95.67	α=96.19	α=95.49
	β=110.4	β=108.38	β=90	β=109.51	β=113.12	β=111.50	β=111.62	β=109.38	β=111.24	β=109.36	β=111.77
	γ=94.5	γ=93.65	γ=90	γ=93.69	γ=93.73	γ=94.28	γ=94.43	γ=94.03	γ=94.05	γ=93.78	γ=94.43
$Fe_4H_4C_4O_{12}$	α=90	α=90	α=90	α=90	α=90	α=90	α=90	α=90	α=90	α=90	α=90
	β=90.6	β=71.88	β=90	β=92.53	β=90	β=90.59	β=90.59	β=90	β=88.68	β=72.30	β=89.75
	γ=90	γ=90	γ=90	γ=90	γ=90	γ=90	γ=90	γ=90	γ=90	γ=90	γ=90
$Fe_4P_4H_{16}C_8O_{24}$	α=90	α=90	α=90	α=90	α=90	α=90	α=90	α=90	α=90	α=90	α=90
	β=90	β=90	β=90	β=90	β=90	β=90	β=90	β=90	β=90	β=90	β=90
	γ=90	γ=90	γ=90	γ=90	γ=90	γ=90	γ=90	γ=90	γ=90	γ=90	γ=90
$Sm_2H_{12}C_{10}O_{14}$	α=101.9	α=103.32	α=101.47	α=101.32	α=99.95	α=100.61	α=102.17	α=101.89	α=101.41	α=103.13	α=101.22
	β=103.1	β=102.78	β=101.62	β=101.89	β=102.29	β=102.21	β=104.11	β=102.32	β=103.13	β=99.57	β=103.25
	γ=101.4	γ=98.21	γ=103.20	γ=103.44	γ=101.87	γ=102.45	γ=99.15	γ=102.87	γ=101.81	γ=97.12	γ=100.86
**MSE**		**-0.38**	**-0.48**	**-0.0005**	**-0.13**	**-0.09**	**0.008**	**0.15**	**-0.07**	**-0.20**	**-0.08**
**MUE**		**0.99**	**1.34**	**0.49**	**0.37**	**0.32**	**0.31**	**0.37**	**0.28**	**1.26**	**0.28**
**MUPA (%)**		**2.38**	**1.35**	**2.25**	**1.52**	**3.52**	**0.32**	**0.40**	**0.29**	**1.36**	**0.29**

Table S9: Lattice constants and band gaps of ten semiconductors calculated with the revM06-L and SCAN functionals

Systems	Experimental[a]		revM06-L		SCAN	
	**Lattice Constants (Å)**	**Band Gaps (eV)**	**Lattice Constants (Å)**	**Band Gaps (eV)**	**Lattice Constants (Å)**	**Band Gaps (eV)**
**Si**	*a*=*b*=*c*= 5.430	1.17	5.430	1.10	5.423	0.95
**Ge**	*a*=*b*=*c*= 5.657	0.74	5.707	1.13	5.687	1.13
**SiC**	*a*=*b*=*c*= 4.348	2.42	4.319	2.35	4.348	1.87
**β-GaN**	*a*=*b*=*c*= 4.545	3.30	4.514	2.61	4.523	2.27
**CdS**	*a*=*b*=*c*= 5.818	2.55	5.909	2.34	5.879	1.68
**CdSe**	*a*=*b*=*c*= 6.213	1.90	6.172	1.67	6.134	1.12
**ZnS**	*a*=*b*=*c*= 5.409	3.66	5.387	3.45	5.375	2.74
**ZnO**	*a*=*b*=3.249 *c*=5.207	3.40	*a*=*b*=3.189 *c*=5.162	1.87	*a*=*b*=3.234 *c*=5.224	1.73
**TiO_2 (anatase)**[b]	*a*=*b*=3.803 *c*=9.748 (18)	3.20 (19)	*a*=*b*=3.802 *c*=9.590	2.56	*a*=*b*=3.797 *c*=9.547	2.27
**TiO_2 (rutile)**[b]	*a*=*b*=4.653 *c*=2.969 (18)	3.00 (20)	*a*=*b*=4.621 *c*=2.961	2.19	*a*=*b*=4.622 *c*=2.951	1.90
**MSE**			–0.01	–0.40	–0.05	–0.77
**MUE**			0.05	0.48	0.04	0.85
**MUPE**			**0.68**	**19.54**	**0.57**	**48.96**

[a]The experimental lattice constants are taken from the LC17 database[21] and the band gaps are taken from the SBG31 database[21] except for the anatase and rutile phases of TiO_2.

[b]The references for the experimental lattice constants and band gaps of TiO_2 (anatase and rutile) are given in the parentheses after the experimental values.

[c]MSE: mean signed error; MUE: mean unsigned error; MUPE: mean unsigned percentage error.

References

1. Frenzer, W.; Wartchow, R.; Bode, H.; Crystal structure of disilver 2,5-dichloro-[1,4]benzoquinone-3,6-diolate, $Ag_2(C_6O_4Cl_2)$. *Zeitschrift Fur Kristallographie,* **1997**, *212*, 237.
2. Tian, Y-Q.; Yao, S.-Y.; Gu, D.; Cui, K.-H.; Guo, D.-W.; Zhang, G.; Chen, Z. X.; Zhao, D.-Y.; Cadmium imidazolate frameworks with polymorphism, high thermal stability, and a large surface area. *Chem. Eur. J.***, 2010,** *16*, 1137-1141.
3. Wang, C. C.; Kuo, C. T.; Yang, J. C.; Lee, G. H.; Shih, W. J.; Sheu, H. S.; Assemblies of two new metal−organic frameworks constructed from Cd(II) with 2,2'-bipyrimidine and cyclic oxocarbon dianions $C_nO_n^{2-}$ (n = 4, 5). *Cryst. Growth Des.* **2007**, *7,* 1476-1482.
4. Bowden, T. A.; Milton, H. L.; Slawin, A. M. Z.; Lightfoot, P.; Hydrothermal syntheses and crystal structures of three zinc succinates: $Zn(C_4H_4O_4)$-α, $Zn(C_4H_4O_4)$-β and $K_2Zn(C_4H_4O_4)_2$. *Dalton Trans.* **2003**, 936-939.
5. Xie, L. H.; Lin, J. B.; Liu, X. M.; Wang, Y.; Zhang, W. X.; Zhang, J. P.; Chen, X. M.; Porous coordination polymer with flexibility imparted by coordinatively changeable lithium ions on the pore surface. *Inorg. Chem.* **2010**, *49*, 1158-1165.
6. Kurmoo, M., Kepert, C. J.; Hard magnets based on transition metal complexes with the dicyanamide anion, $\{N(CN)_2\}^-$. *New J. Chem.*, **1998**, *22*, 1515-1524.
7. Cao, R.; Shi, Q.; Sun, D. F.; Hong, M. C.; Bi, W. H.; Zhao, Y. J.; Syntheses and characterizations of Copper(II) polymeric complexes constructed from 1,2,4,5-benzenetetracarboxylic acid. *Inorg. Chem.* **2002***, 41*, 6161-6168.
8. Ouellette, W.; Prosvirin, A. V.; Chieffo, V.; Dunbar, K. R.; Hudson, B.; Zubieta, J.; Solid-state Coordination Chemistry of the Cu/triazolate/X System (X = F-, Cl-, Br-, I-, OH-, and $SO_4(^{2-})$). *Inorg. Chem.* **2006**, *45,* 9346-9366.
9. Kong, X. J.; Zhuang, G. L.; Ren, Y. P.; Long, L. S.; Huang, R. B.; Zheng, L. S.; In situ cyclodehydration of iminodiacetic acid into 2,5-diketopiperazine-1,4-diacetate in lanthanide-based coordination polymers. *Dalton Trans.* **2009**, 1707-1709.
10. Molinier, M.; Price, D. J.; Wood, P. T.; Powell, A. K.; Biomimetic control of iron oxide and hydroxide phases in the iron oxalate system. *J. Chem. Soc., Dalton Trans*., **1997**, 4061-4068.
11. Hou, J. J., Zhang, X. M.; Structures and magnetic properties of a series of metal phosphonoacetates synthesized from in situ hydrolysis of triethyl phosphonoacetate. *Cryst. Growth Des*. **2006**, *6*, 1445-1452.
12. Zhang, X. J.; Xing, Y. H.; Han, J.; Zeng, X. Q.; Ge, M. F.; Niu, S. Y.; A series of novel ln−succinate−oxalate coordination polymers: synthesis, structure, thermal stability, and fluorescent properties. *Cryst. Growth Des*. **2008**, 8, 3680-3688.
13. Cavka, J. H.; Jakobsen, S.; Olsbye, U.; Guillou, N.; Lamberti, C.; Bordiga, S.; Lillerud, K. P. A New zirconium inorganic building brick forming metal organic frameworks with exceptional stability. *J. Am. Chem. Soc.* **2008**, *130*, 13850-13851.
14. Hailian Li, Mohamed Eddaoudi, M. O'Keeffe & O. M. Yaghi, Design and synthesis of an exceptionally stable and highly porous metal-organic framework. *Nature*, **1999**, *402*, 276-279.

15. Martin, R. L.; Smit B.; Haranczyk, M.; Addressing challenges of identifying geometrically diverse sets of crystalline porous materials. *J. Chem. Inf. Model.* **2012**, *52*, 308-318.
16. Willems, T. F.; Rycro, C.; Kazi, M.; Meza, J. C.; Haranczyk, M.; Algorithms and tools for high-throughput geometry- based analysis of crystalline porous materials, Microporous and Mesoporous Materials. **2012**, *149*, 134-141.
17. Pinheiro, M.; R. L. Martin, C. H. Rycro, A. Jones, E. Iglesia, and M. Haranczyk, Characterization and comparison of pore landscapes in crystalline porous materials. *J. Mol. Graphics Modell.* **2013,** *44*, 208-219.
18. Wyckoff. R. W. G. Crystal Structures; second edition; Interscience Publishers: New York, **1963**, Vol. **1**, p. 239.
19. Rahimi, N. v.; Pax, R. A., Gray, E. M., Review of functional titanium oxides. I: TiO_2 and its modifications. *Solid State Chem*. **2016,** *44*, 86-105.
20. Noda, L. K.; Rosales, R.; Goncalves, N. S.; Sala, O.; Evidences for charge-transfer complex formation in the benzene adsorption on sulfated TiO_2–a resonance Raman spectroscopy investigation. *J. Raman Spectrosc*. **2008**, *39*, 415-420.
21. Peverati, R.; Truhlar, D. G.; Performance of the M11-L Density Functional for Bandgaps and Lattice Constants of Unary and Binary Semiconductors. *J. Chem. Phys.* **2012,** *136*, 134704.
